# Effects of an educational intervention with nursing professionals on approaches to hospitalized smokers: a quasi-experimental study*

**DOI:** 10.1590/1980-220X-REEUSP-2021-0569en

**Published:** 2022-05-13

**Authors:** Fernanda Guarilha Boni, Yasmin Lorenz da Rosa, Renata Meirelles Leite, Fernanda Machado Lopes, Isabel Cristina Echer

**Affiliations:** 1 Universidade Federal do Rio Grande do Sul, Escola de Enfermagem, Programa de Pós-Graduação em Enfermagem, Porto Alegre, RS, Brazil.; 2 Universidade Federal do Rio Grande do Sul, Escola de Enfermagem, Porto Alegre, RS, Brazil.; 3 Universidade Federal de Santa Catarina, Departamento de Psicologia, Florianópolis, SC, Brazil.

**Keywords:** Tobacco Use Cessation, Health Education, Nursing Team, Education, Nursing, Continuing, Teaching, Cese del Uso de Tabaco, Educación en Salud, Grupo de Enfermería, Educación Continua en Enfermería, Enseñanza, Abandono do Uso de Tabaco, Educação em Saúde, Equipe de Enfermagem, Educação Continuada em Enfermagem, Ensino

## Abstract

**Objective:**

to assess the effects of an educational intervention on smoking cessation aimed at the nursing team.

**Method:**

this is a quasi-experimental study with 37 nursing professionals from a Brazilian hospital from May/2019 to December/2020. The intervention consisted of training nursing professionals on approaches to hospitalized smokers divided into two steps, the first, online, a prerequisite for the face-to-face/videoconference. The effect of the intervention was assessed through pre- and post-tests completed by participants. Smokers’ medical records were also analyzed. For analysis, McNemar’s chi-square test was used.

**Results:**

there was an increase in the frequency of actions aimed at smoking cessation after the intervention. Significant differences were found in guidelines related to disclosure to family members of their decision to quit smoking and the need for support, encouragement of abstinence after hospital discharge, and information on tobacco cessation and relapse strategies.

**Conclusion:**

the educational intervention proved to be innovative and with a great capacity for disseminating knowledge. The post-test showed a positive effect on the frequency of actions aimed at smoking cessation implemented by the nursing team.

## RESUMO

**Objetivo:**

avaliar os efeitos de uma intervenção educativa sobre cessação do tabagismo direcionada à equipe de enfermagem.

**Método:**

estudo quase-experimental com 37 profissionais de enfermagem de um hospital brasileiro de maio/2019 a dezembro/2020. A intervenção consistiu em capacitar profissionais de enfermagem sobre abordagens aos pacientes tabagistas, dividida em duas etapas, a primeira, *online*, pré-requisito para a presencial/videoconferência. O efeito da intervenção foi avaliado por meio do pré- e pós-teste preenchido pelos participantes. Também foram analisados registros em prontuários de pacientes fumantes. Para análise, utilizou-se o Teste do Qui-Quadrado de McNemar.

**Resultados:**

houve aumento da frequência das ações visando à cessação tabágica após a intervenção. Diferenças significativas foram encontradas em orientações relacionadas à divulgação aos familiares da decisão de parar de fumar e necessidade de apoio, incentivo à abstinência após alta hospitalar e informações sobre estratégias para cessação do tabaco e recaídas.

**Conclusão:**

a intervenção educativa se mostrou inovadora e com grande capacidade de difusão do conhecimento. O pós-teste evidenciou efeito positivo na frequência das ações visando à cessação tabágica implementadas pela equipe de enfermagem.

## RESUMEN

**Objetivo:**

evaluar los efectos de una intervención educativa para dejar de fumar dirigida al equipo de enfermería.

**Método:**

estudio cuasi-experimental con 37 profesionales de enfermería de un hospital brasileño de mayo/2019 a diciembre/2020. La intervención consistió en capacitar a los profesionales de enfermería en el abordaje del paciente fumador, dividida en dos etapas, la primera, en línea, requisito previo para la presencial/videoconferencia. El efecto de la intervención se evaluó a través del pre y post test realizado por los participantes. También se analizaron los registros en las historias clínicas de los fumadores. Para el análisis se utilizó la prueba Chi-Square de McNemar.

**Resultados:**

hubo un aumento en la frecuencia de acciones dirigidas a dejar de fumar después de la intervención. Se encontraron diferencias significativas en las guías relacionadas con la divulgación a los familiares de la decisión de dejar de fumar y la necesidad de apoyo, el estímulo de la abstinencia después del alta hospitalaria y la información sobre estrategias para dejar de fumar y recaer.

**Conclusión:**

la intervención educativa demostró ser innovadora y con gran capacidad de diseminación del conocimiento. El post-test mostró un efecto positivo en la frecuencia de las acciones dirigidas a la deshabituación tabáquica implementadas por el equipo de enfermería.

## INTRODUCTION

High hospital admission rates for smokers highlight the need to qualify the care provided by health professionals in order to implement effective approaches to smoking cessation^(1)^. It has already been shown that smokers newly diagnosed with a chronic disease were more sensitized to change their behavior^(2–3)^ and that concern for their own health is one of the main reasons that lead people to quit smoking^(4)^, indicating that admission time seems favorable to guidance, education and awareness of tobacco cessation actions.

Considering that the nursing team is constantly in direct contact with patients, it is important that they are able and qualified to provide quality care to hospitalized smokers^(5)^. However, professionals still feel unprepared to carry out this approach, which may be related to the lack of specific skills and knowledge on the subject, work overload, disregard of this practice as their attribution or little incentive from their health institution^(6–7)^. A study that analyzed medical records of 69 hospitalized smokers found that only 48% of the anamnesis had a smoking status; of these, only 13% included smoking time and the number of cigarettes smoked daily, demonstrating the need to qualify care and records^(8)^.

A survey of nurses who carried out an online training on assistance to hospitalized smokers with a view to smoking cessation showed that, after completing the course, referrals of smokers to treatment increased significantly. However, the authors suggested that adding a face-to-face step to complement the virtual one could have an even greater positive impact, as well as have a more lasting change in care practices^(9)^.

From this perspective, this study aimed to assess the effects of an educational intervention on smoking cessation aimed at the nursing team. Its realization is justified, since smoking continues with worrying prevalence rates and the implementation of actions to change this reality are always valuable, as they can add efforts to reduce this serious problem of global public health.

## METHOD

### Design of Study

This is a quasi-experimental, non-randomized study, based on pre- and post-intervention that analyzed the effects related to the approach to smoking patients aiming at smoking cessation. In this type of research, individuals are their own control before and after the intervention^(10)^.

This research proposed assessing an educational intervention entitled “Training on approaches to promote smoking cessation in hospitalized patients”, developed and validated by experts on the subject^(11)^. It addresses issues related to smoking, such as: definition, related diseases, chemical and behavioral dependence; decision to quit smoking; approaches at patient admission, during hospitalization, at discharge and post-discharge; steps of readiness for change; strategies to quit smoking; benefits of quitting smoking; medication use; ­monitoring of smokers; and the institution’s tobacco control plan. As a teaching and learning method, the blended approach was used, which consists of a step carried out in the distance learning format complemented by a face-to-face session carried out at the institution and lasting one hour and 30 minutes. In this step, the topics covered in the distance education format and exchange of experiences between professionals were carried out. Participants received scales to measure nicotine ­dependence and motivation to change behavior, books and folders to ­support approaches to patients. However, as of March 2020, as a result of the Coronavirus Disease 2019 (COVID-19) pandemic, these face-to-face meetings began to be held remotely and synchronously by videoconference. 

### Population

The population consisted of nursing professionals from clinical and surgical units who participated in a course on approaches to hospitalized smokers. 

### Local

This research was carried out from May 2019 to December 2020 in a large university hospital in southern Brazil, which serves a mean of 1,529 hospitalized patients per month and has 2,796 nursing professionals.

### Selection Criteria

The sample included nursing professionals who agreed to respond to an evaluative activity before and after three months of course. All professionals were invited to participate in the training, with no exclusion criteria. Those who performed the educational intervention, but who went on leave/vacation during the data collection period, were considered losses. 

### Data Collection

To assess the educational intervention effects with nursing professionals, the researchers developed an instrument in a previous study^(11)^, which was validated by experts and subsequently submitted to a pilot test for refinement and adequacy. With the authors’ prior authorization, this instrument was made available on Google Forms^®^ and sent by email before and after participating in the training.

**Figure 1:**
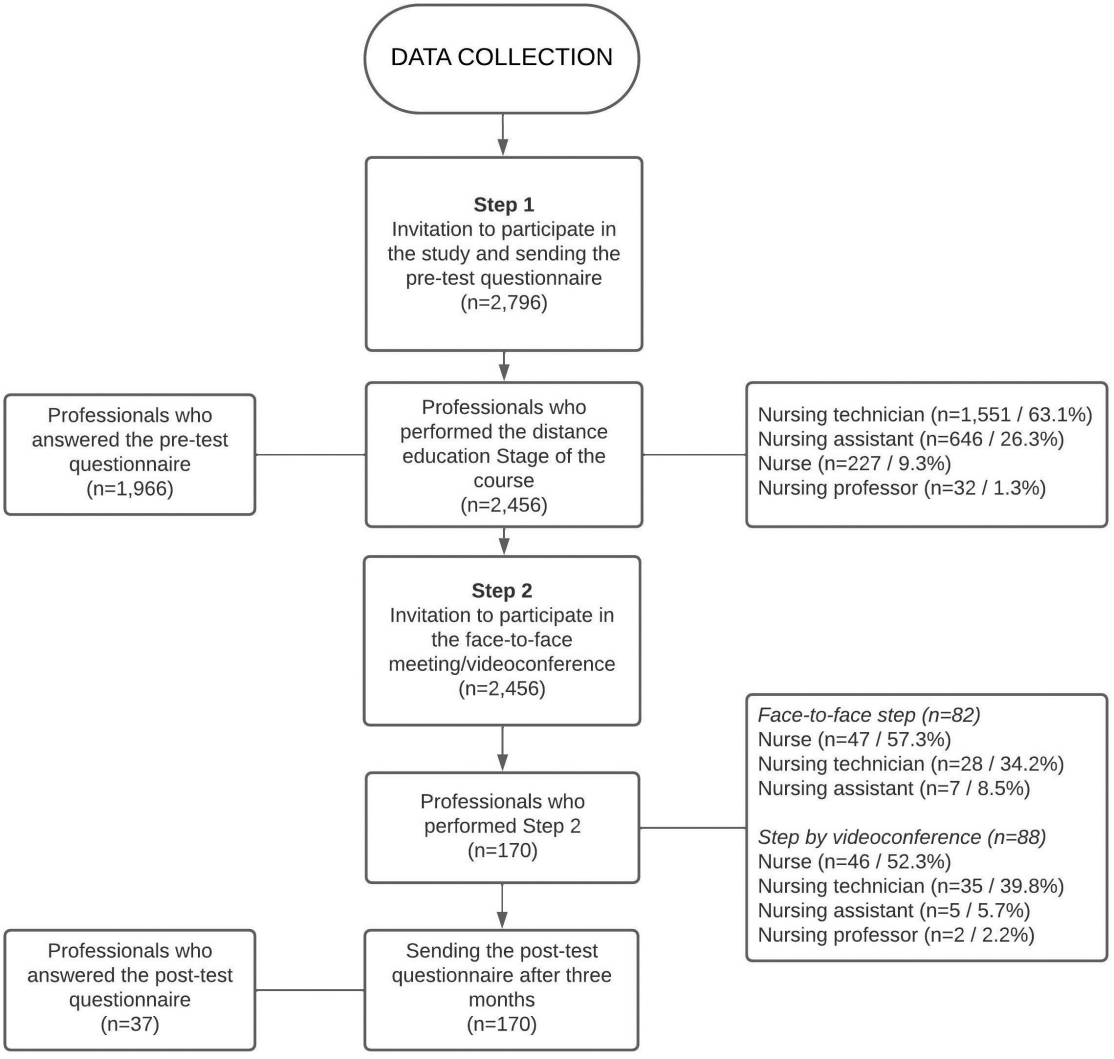
Data collection flowchart.

The first part of the form had six questions related to professional characterization, and the second, 10 questions about actions considered essential in approaching the smoking patient aiming at cessation. The frequency of these actions was self-reported and classified as “Always”, “Sometimes” and “Never”. The mean time to complete the questionnaire was seven minutes. The flowchart in Figure 1 shows how data collection took place, as well as the number of invited professionals who participated in the study.

At the end of the period for sending the forms for the post-test, the electronic medical records of all patients over 18 years old hospitalized in clinical and surgical units of the institution where this study was carried out were accessed. The choice of these units is due to the fact that a previous study has already been carried out in which the smoking status of patients hospitalized in these places was analyzed^(8)^. The purpose was to verify whether, in addition to the activities common to the nursing team, the records made privately by nurses about patients’ smoking status were being carried out properly in the anamnesis and whether the nursing prescription contained specific nursing diagnoses and care for smokers. 

### Data Analysis and Treatment

Data analysis was obtained from participants’ responses to pre- and post-test questionnaires. Continuous variables were described by mean and standard deviation for those with normal distribution or median and interquartile range when asymmetric. To compare the pre and post intervention, the generalized version of McNemar’s chi-square test was used. The analyzes were performed using the Statistical Package for the Social Sciences (SPSS) version 23.0 program.

Regarding secondary data, collected from smokers’ electronic medical records, the statistical power of the test was assessed to compare the proportions of correct recording of the anamnesis of these individuals. It was considered that 69 hospitalized smokers would be assessed in November and December 2020. Taking into account that before the intervention, in August and September 2017, the proportion of correct anamnesis records was 13%^(8)^, and assuming that this percentage would increase to 50% as a relevant effect of the educational intervention, it was calculated that the power of the test would be 99%. Thus, it was estimated to assess about 414 medical records until reaching, at least, 69 smokers. The power calculation was performed using WinPEPI^®^ 11.43.

### Ethical Aspects

This study met the ethical requirements of research involving human beings, and was approved by the Research Ethics Committee of the institution on May 8, 2019, under opinion number 3,313,795, obtaining CAAE (*Certificado de Apresentação para Apreciação Ética* – Certificate of Presentation for Ethical Consideration) 64475916700005327.

Regarding the instruments that professionals answered anonymously via Google Forms^®^, there was the following ­information in the body of the text for consent to participate in the study: *by answering it, you will be agreeing to the use of data in the research. Anonymity will be guaranteed and your participation will make it possible to improve the course on the approach to hospitalized smokers.*

## RESULTS

The pre-test questionnaire was answered by 1,966 participants and, of these, 37 completed the post-test. Considering only those who responded to the assessments before and after the educational intervention, 37 nursing professionals participated in the study, most of them women (n = 34; 91.9%), with a mean age of 47.1 ± 7.6 years and 23.1 ± 7.7 years of job tenure. Regarding occupation, 25 (67.6%) were nurses and with regard to smoking profile, only one (2.7%) professional reported being a smoker. This and other information about participant characterization is described in Table 1. 

**Table 1:** Sample characterization (n = 37) – Porto Alegre, RS, Brazil, 2021.

**Variables analyzed**	**N (%)**
**Sex**	
Female	34 (91.9%)
Age*	47.1 ± 7.6
**Professional category**	
Nursing technician	12 (32.4%)
Nurse	25 (67.6%)
Job tenure in years*	23.1 ± 7.7
**Education level**	
High school	10 (27%)
Undergraduate education	1 (2.7%)
Specialization	14 (37.8%)
Master’s degree	9 (24.3%)
Doctoral degree	3 (8.1%)
**Smoking status**	
Smoker	1 (2.7%)
Smoking time in years	1
Packs consumed per year	18
Non-smoker	34 (91.9%)
Non-smoker, but lives with smokers	2 (5.4%)

*Result presented as mean ± standard deviation.

Before performing the educational intervention, 15 (40.5%) of 37 professionals felt prepared to approach hospitalized smokers. After carrying out the two steps proposed in training, 27 (73%) reported feeling ready. This and other information about the frequency of actions aimed at smoking cessation implemented by the nursing team are described in Table 2.

**Table 2:** Frequency of actions aimed at smoking cessation implemented by nursing professionals before and after the educational intervention (n = 37) – Porto Alegre, RS, Brazil, 2021.

**Variables assessed**	**Before intervention**	**After intervention**	**p**
You feel prepared to approach hospitalized smokers			0.065
*Yes*	15 (40.5%)	27 (73%)	
*No*	5 (13.5%)	2 (5.4%)	
*Partially*	17 (45.9%)	8 (21.6%)	
Assesses whether patients are motivated to quit smoking			0.179
*Always*	13 (35.1%)	21 (56.8%)	
*Sometimes*	20 (54.1%)	15 (40.5%)	
*Never*	4 (10.8%)	1 (2.7%)	
Addresses patients about smoking cessation during hospitalization			0.062
*Always*	12 (32.4%)	23 (62.2%)	
*Sometimes*	20 (54.1%)	11 (29.7%)	
*Never*	5 (13.5%)	3 (8.1%)	
Relates the reason for the current hospitalization with smoking			0.064
*Always*	13 (35.1%)	23 (62.2%)	
*Sometimes*	17 (45.9%)	13 (35.1%)	
*Never*	7 (18.9%)	1 (2.7%)	
Guides smoking cessation strategies and provides support material (folders/manuals)			0.272
*Always*	10 (27%)	11 (29.7%)	
*Sometimes*	18 (48.6%)	22 (59.5%)	
*Never*	9 (24.3%)	4 (10.8%)	
Advises patients to set a date to quit smoking			0.170
*Always*	6 (16.2%)	10 (27%)	
*Sometimes*	14 (37.8%)	19 (51.4%)	
*Never*	17 (45.9%)	8 (21.6%)	
Guides on difficulties related to abstinence symptoms			0.672
*Always*	15 (40.5%)	15 (40.5%)	
*Sometimes*	14 (37.8%)	17 (45.9%)	
*Never*	8 (21.6%)	5 (13.5%)	
Encourages the disclosure to family members of their decision to quit smoking and the need for support			0.028
*Always*	7 (18.9%)	16 (43.2%)	
*Sometimes*	14 (37.8%)	16 (43.2%)	
*Never*	16 (43.2%)	5 (13.5%)	
Guides and encourages tobacco abstinence after hospital discharge			0.033
*Always*	14 (37.8%)	27 (73%)	
*Sometimes*	17 (45.9%)	8 (21.6%)	
*Never*	6 (16.2%)	2 (5.4%)	
Guides that relapses are part and that patients should analyze strategies to quit smoking			0.006
*Always*	9 (24.3%)	21 (56.8%)	
*Sometimes*	16 (43.2%)	13 (35.1%)	
*Never*	12 (32.4%)	3 (8.1%)	

Source: research data, 2021.

When comparing participants’ means before and after the educational intervention, significant differences were found in the behavior of “encouraging the disclosure to family members of their decision to quit smoking and the need for support”, “guiding and encouraging tobacco abstinence after hospital discharge” and “guiding that relapses are part and that patients should analyze strategies to quit smoking”. In addition to these, it can be observed that there was a tendency to change (with p close to 0.06) in the behaviors of “approaching patients about smoking cessation during hospitalization” and “relating the reason for the current hospitalization with smoking” and also how prepared they felt to approach hospitalized smokers.

In order to identify which actions are being recorded, the electronic records of 1,128 patients admitted and assisted by nursing professionals who took the course were also analyzed. Among the patients, 604 (53.5%) were male with a mean age of these patients was 58.2 ± 17.4 years and the median number of days of hospitalization was 8.2 (4.1–15). Regarding participants’ smoking status, 80 (7.1%) were smokers and 85 (7.5%) smokers were smokers in abstinence. This and other information are described in Table 3.

**Table 3:** Data obtained from smokers’ electronic medical records (N = 1,128) – Porto Alegre, RS, Brazil, 2021.

**Variables assessed**	**N (%)**
**Sex**	
Male	604 (53.5%)
Age in years*	58.2 ± 17.4
**Hospital stay in days** ^†^	8.2 (4.1–15)
***Smoking* status**	
Smoker	80 (7.1%)
Smoking time in years*	33.9 ± 15.4
*Cigarettes consumed per day* ^†^	20 (10–27.5)
Smoker in abstinence	85 (7.5%)
*Time without smoking in years* ^†^	10.5 (3–20.7)
Non-smoker	940 (83.3%)
No medical records	23 (2%)

*Result presented as mean ± standard deviation; ^†^Resulting in median (P25–P75). Source: research data, 2021.

Finally, the reason for hospitalization of smokers and smokers in abstinence was assessed, and biopsy/staging and diagnosis of neoplasia/cancer surgery were the most frequent causes (34; 20.6%). Moreover, it was also verified whether a smoking status record was correctly and completely filled out in the anamnesis and whether any specific nursing diagnosis for this condition was included in the prescription (Table 4).

**Table 4:** Assessment of nursing records in electronic medical records of smokers and smokers in abstinence (N = 165) – Porto Alegre, RS, Brazil, 2021.

**Variables assessed**	**N (%)**
Reasons for hospitalization	
*Biopsy/staging and diagnosis of neoplasia/oncologic surgery/chemotherapy*	34 (20.6%)
*By-pass/vascular surgery/arteriography*	22 (13.3%)
*Chemical dependence*	12 (7.3%)
*Abdominal pain/weight loss/diarrhea/vomiting*	11 (6.7%)
*Orthopedic surgery*	11 (6.7%)
Correct record in anamnesis in relation to tobacco use	73 (44.2%)
Presence of Nursing Diagnosis related to substance abuse	9 (4.9%)
*Risk-prone health behavior*	5 (62.5%)
*Risk of acute substance abstinence syndrome*	2 (25%)
*Acute substance abstinence syndrome*	1 (12.5%)
Nursing care prescription	11 (6%)
*Assists patients to identify realistic and attainable goals*	3
*Encourages treatment compliance*	1
*Assesses verbal and nonverbal signs of anxiety*	1
*Provides calm and comfortable environment*	1
*Communicates abstinence signals*	1
*Communicates anxiety indicator behavior*	1
*Reinforces important information*	1
*Encourages patients to develop control and responsibility over their own treatment*	2

Source: research data, 2021.

## DISCUSSION

Compliance with the educational intervention in a blended modality on approaches to promote smoking cessation in hospitalized patients was significant, which highlights the concern of nursing professionals in acquiring skills to assist smokers. However, there was a greater participation of those enrolled in the training step carried out in the distance education modality (n = 2,456) when compared to the face-to-face/synchronous videoconference step (n = 170), and the engagement of professionals in completing the post-test questionnaire was low (n = 37). 

A possible explanation for the lower participation in the face-to-face or videoconference step is that the field site of this study has become a reference hospital for the care of patients infected by COVID-19, which generated work overload, displacement of teams in the institution and hiring of newly graduated professionals. Despite this, the professionals who participated in the intervention of this study assessed it positively and considered it relevant.

Faced with atypical situations that health institutions have been experiencing as a result of the coronavirus pandemic, the online teaching modality introduction has proved to be relevant^(12)^. Specifically in the context of continuous training of professionals about smoking, isolated strategies of distance education have been shown to be effective in terms of the teaching-learning process^(13,14,15)^, since it is an important tool for raising awareness of the nursing team on the subject^(11)^. 

With technological innovation, access to information becomes easier, allowing professionals to search for current and quality knowledge to offer the best care to patients. In this sense, courses offered by the institutions themselves, such as the qualification for approaching smokers^(11)^ provided by the field in which the present study was carried out, are examples of learning tools that can contribute to the improvement of care practice. 

The literature indicates that educational interventions with the nursing team regarding smoking cessation provide information, encourage approaches and improve their effectiveness^(16,17,18)^. The fact that not all participants in the present study reported that they felt able to approach smokers after taking the course reveals that completing the training activity does not necessarily guarantee ability to develop the content learned in care practice. In addition, not all trained individuals will become tobacco experts, but it is critical that they acquire sufficient knowledge to carry out interventions and understand how nicotine dependence works, the possibilities of treatment and the support that needs to be offered^(19)^. Thus, it is suggested that systematic debates and clinical studies be carried out among professionals on the subject in order to qualify their approaches to patients.

Regarding the effects of applying the educational intervention in the present research, the comparative results (pre- and post-test) of the frequency of actions to promote smoking cessation implemented by the nursing team suggest that there was a better assessment of their skills after the intervention, although this difference was not significant in all items. The findings showed that the increase in actions was about informing family members of their decision to quit smoking and the need for support, encouraging abstinence after hospital discharge, and providing guidance on strategies to avoid relapses. The frequency of other actions, such as approaching patients about smoking cessation, relating the reason for the current hospitalization to smoking, and feeling prepared to approach the smoking patient also increased. However, the difference between the pre- and post-assessments only touched a significance, a result that could have greater statistical relevance with the increase in the sample. These results elucidate the importance of discussing this topic in hospital institutions and the need for teams to receive training to carry out effective approaches. 

Corroborating this conception, an American health institution, realizing the little time spent by nurses with patients to quit smoking, created a smoking cessation program to prioritize focused and instrumental care for cancer patients, performed by specialized professionals trained by the program itself^(20)^. Considering the limited availability of time for professionals to properly carry out smoking cessation activities during hospitalization, it is believed that creating groups of experts prepared to provide consultations aimed at smoking cessation can be an important strategy for a greater number of patients have access to adequate support of information about physical, psychological and behavioral dependence on tobacco and about the provision of devices that relieve abstinence symptoms.

Another strategy that can change this reality is the inclusion of the tobacco issue in university curricula through evidence-based methods. A cohort study carried out in Canada with medical students from nine universities showed that there is still a lack of training and capacity building for specific approaches to smoking^(21)^. In this perspective, studies reinforce that the training of professionals to approach smokers should start at graduation and include all courses in the health area^(22,23,24,25)^, including the standardization of curricula to address this subject^(21)^.

Although the findings of this study have pointed to an increase in the percentage of nursing records performed correctly, identified as 13% in a previous study^(8)^, the 50% considered as a relevant effect of the intervention was not reached. These data are in line with other studies that show that low compliance with interventions and medical records by health professionals is recurrent^(7,19,26)^. This scenario reinforces the relevance of training on the subject and the importance of continuing to occur continuously during care practice and with proposals to discuss clinical cases involving smokers.

In academia, the negative impact that smoking has on health has been widespread education, mainly because it is the main cause of preventable death^(27)^. It is noteworthy that, although the reasons for hospitalization analyzed in this study were due to tobacco-related diseases, this was not reflected in actions recorded by health professionals in relation to smoking cessation. These results are worrying, as hospitalization is an important opportunity to motivate smokers to quit smoking and to promote healthy lifestyle habits.

The results of this study did not show a significant change in relation to the use of some educational resources to guide patients about smoking cessation. However, it is reiterated the importance of alerting that approaching patients about the need to quit smoking requires persistence, knowledge and time to carry out this action in a cautious way, since it is a delicate subject for most patients. 

In this perspective, the construction and implementation of materials such as manuals and institutional folders to support health guidelines can be an effective strategy. Corroborating this thought, a study that sought to assess the factors associated with interventions performed by nurses aimed at smoking cessation identified that, in addition to knowledge and skills, confidence in this type of assistance, time availability, being able to take the necessary interventions to quit smoking as a responsibility of work and support of the unit are important issues that imply compliance of professionals to the approaches^(7)^. 

In this context, it is clear that not considering smoking cessation as a priority activity, as is the case of professionals with intense demands and a low contingent of workers in the team of inpatient units, negatively influences the frequency of interventions. However, carrying out systematic training with the nursing team can achieve positive results and even increase the importance that professionals give to their involvement in tobacco control, even when compared to actions related to other pathologies^(5)^.

## CONCLUSION

The findings of this research show that there was an increase in the frequency of actions aimed at smoking cessation after the intervention and also demonstrate the interest of nursing professionals in carrying out training on this topic, since the number of participants was significant. Furthermore, as a product, this study allowed the implementation of new routines for approaching and recording the medical records of smokers in the researched institution. The synchronous holding of a face-to-face and/or videoconference meeting, after the distance education step, has potentiated the reflection and discussion of professionals on the subject by enabling the exchange of experiences and guidance for handling specific situations.

The main limitation is the low compliance of professionals with returning the post-test forms. This fact may have occurred because this research was carried out during the pandemic period in a reference hospital for the care of patients with COVID-19. However, the frequency of actions aimed at smoking cessation implemented by nursing professionals show that there was a positive effect on the assessment of their skills in the ­post-test. As a limitation, there is the fact that this study does not have parameters for comparison in relation to smokers, but it ­carried out a verification of practical application, in addition to having been conducted in a single center, not allowing the generalization of the present findings. 

Finally, it is expected that the results of this study will serve as inspiration for new strategies to be developed that include other areas of the multidisciplinary team, because all health professionals have a responsibility to approach and educate smokers about this serious health problem.

## ASSOCIATE EDITOR

Cristina Lavareda Baixinho
